# Evaluation of a Curriculum-Based Nutrition Education Intervention Protocol in Elementary Schools: Nonrandomized Feasibility Study

**DOI:** 10.2196/69242

**Published:** 2025-04-16

**Authors:** Jacqueline Marie Brown, Nicholas Rita, Beatriz Franco-Arellano, Ann LeSage, Joanne Arcand

**Affiliations:** 1 Faculty of Health Sciences, Ontario Tech University, 2000 Simcoe Street North, Oshawa, ON, L1G 0C5, Canada, 1 9057218668; 2 Mitch and Leslie Frazer Faculty of Education, Ontario Tech University, Oshawa, ON, Canada

**Keywords:** nutrition education, serious games, children, food literacy, school nutrition intervention, feasibility

## Abstract

**Background:**

Improving children’s food literacy through school-based interventions can support developing healthy eating habits. However, teachers lack appropriate resources, time, and training to provide nutrition education in schools. Serious games, which are games designed for a purpose other than entertainment, have been demonstrated to improve children’s food literacy and dietary intake and can address the barriers teachers face in providing nutrition education. Foodbot Factory (Arcand Lab) is a nutrition education intervention that is aligned with curricula and uses a serious game to provide nutrition education to students. Further evidence is needed to understand how serious games, including Foodbot Factory, can be researched in schools to support nutrition education.

**Objective:**

The objective of this study was to evaluate the feasibility of a research study protocol that implements the curriculum-based nutrition education intervention Foodbot Factory into a real-world classroom setting. The evaluation of the protocol included study processes, resources, and management feasibility outcomes, as well as a preliminary assessment of scientific outcomes relevant to the intervention.

**Methods:**

A nonrandomized study determined the feasibility of intervention implementation. Grade 4 and 4/5 classrooms were assigned to have nutrition education lessons for 5 days with either the Foodbot Factory or a control intervention. Outcomes were assessed in 4 feasibility domains of study processes (eg, recruitment and attrition rates), resources (eg, time taken to deliver the intervention), and management (eg, challenges with intervention delivery), and a preliminary assessment of scientific outcomes pertaining to the acceptability and impacts of the interventions. These outcomes were captured in semistructured field notes completed by study staff and a Nutrition Attitudes and Knowledge questionnaire and acceptability questionnaire completed by participants. Data were analyzed descriptively and using a paired *t* test to assess within-group changes in nutrition knowledge.

**Results:**

In total, 4 classrooms participated in the feasibility study, with varying recruitment rates for schools (3/20, 15%), classrooms (4/4, 100%), parents (54/102, 53%), and children (49/54, 91%). The time required to implement the research protocol, including data collection and lesson plans, was sufficient and management of the intervention implementation was overall successful. Some challenges were experienced with classroom management during data collection, specifically with electronic data collection. After the intervention, participants reported a positive affective experience (26/41, 63%) and learning something new about healthy eating (31/41, 76%). Participants in both study groups improved their nutrition knowledge, but the changes were not statistically significant. The Foodbot Factory group had a statistically significant improvement in their knowledge of vegetables and fruit (*P*=.04) and protein foods (*P*=.03).

**Conclusions:**

These findings indicate that the study protocol is feasible to implement and evaluate Foodbot Factory in a representative sample with select modifications to improve recruitment and data collection procedures.

## Introduction

One way to support children in acquiring and maintaining healthy eating patterns is by developing their food literacy. Food literacy describes the set of interrelated food and nutrition knowledge (ie, understanding food groups and the nutrients in foods), food skills (ie, ability to prepare foods and read recipes), and attributes (eg, self-efficacy and confidence) that interact with our broader socioecological environment to shape our dietary behaviors [1]. Research has demonstrated that children and adolescents with higher levels of nutrition knowledge, a core component of food literacy, are more likely to have a higher quality dietary pattern [2,3]. Improving food literacy is especially relevant in Canada where the average child exceeds recommended intakes for saturated fat, sodium and free sugars, and consumes 21%‐25% of their daily caloric intake from foods that are not recommended by dietary guidelines [4,5]. This dietary pattern can increase the future risk of noncommunicable diseases such as cardiovascular disease and type 2 diabetes [6].

School curriculum, policies, and programming are an important way to support the development of child food literacy and healthy eating behaviors. All jurisdictions in Canada have food and nutrition as a core component of elementary health curricula [7], however, teachers face several barriers to implementing nutrition education in their classrooms due to a lack of training and dedicated time and resources for nutrition [8-10]. Published literature shows that well-designed curriculum-based interventions can effectively improve nutrition knowledge and behaviors [11]. A meta-analysis of school- and curriculum-based nutrition education interventions found that experiential learning approaches, such as school gardens and cooking classes, had the greatest impact on child nutrition knowledge and dietary intake [12]. While these experiential teaching approaches enhance the student learning experience, they are resource- and time-intensive and fail to address the needs of teachers. Thus, alternative experiential learning approaches are needed that can effectively support children’s food literacy development and address the teacher-reported barriers to providing nutrition education.

Technology-based nutrition interventions can address some of these teacher-reported barriers, due to high access and ease of use in the classroom [13]. Serious games, which are games designed for a primary reason other than entertainment, have emerged as a leading technology-based educational platform as they use an experiential learning approach [14]. Research on nutrition-focused serious games has found that they can improve vegetable and fruit intake and nutrition knowledge among children [15-17]. Unfortunately, these nutrition-focused games are often not available to the public [18], do not consistently align with relevant nutrition curriculum, and are underinvestigated in classrooms as a resource to support curriculum implementation. Research and resources are required to support teachers in providing nutrition education and create opportunities for children to develop their nutrition knowledge and food literacy in the classroom.

The Foodbot Factory intervention was developed by an interdisciplinary team of dietitians, game developers, teachers, and researchers, to support elementary teachers with curriculum-based nutrition education and to improve children’s nutrition knowledge [19]. The intervention includes a serious game for students played on a mobile app, with lesson plans for teachers. The content is designed for children ages 8‐12 years (Grades 4 and 5) and aligns with the 2019 Ontario Health and Physical Education curriculum and Canada’s Food Guide [20,21]. Our previous proof-of-concept research demonstrated that the Foodbot Factory serious game significantly improved children’s nutrition knowledge compared with a control food-themed game [22]. However, this study was conducted in a controlled setting, not in the intended classroom environment. The lesson plans developed have also not yet been tested in classrooms, which is critical to understand their implementation [23]. Before embarking on a larger randomized trial to evaluate the impact of Foodbot Factory in classrooms, a setting that comes with unique implementation challenges, a feasibility assessment of an intended research protocol is warranted. Therefore, the objective of this research was to determine the feasibility of a research protocol to evaluate the Foodbot Factory intervention and a control intervention with children in the classroom setting. Study processes, resources, management, and scientific outcomes were the feasibility elements assessed. Such data will inform future research protocols to evaluate the efficacy of Foodbot Factory as part of a randomized trial.

## Methods

### Study Design

This was a nonrandomized study to assess the feasibility of implementing a trial protocol for a nutrition education intervention (Foodbot Factory) and a control intervention among Grade 4, 4/5, and 5 elementary school classrooms, over a 5-day period. Classrooms were assigned in a 1:1 ratio to one of two groups: (1) the Foodbot Factory group receiving nutrition education using the Foodbot Factory serious game and lesson plans or (2) the control group receiving nutrition education using nontechnology based learning activities. This study protocol was cocreated with several school board partners. This approach increased the feasibility of the study protocol from the school board’s perspective, particularly in relation to the length of time required for the study and the use of a study teacher to provide the intervention (described in the Interventions: Overview section).

**Figure 1.**
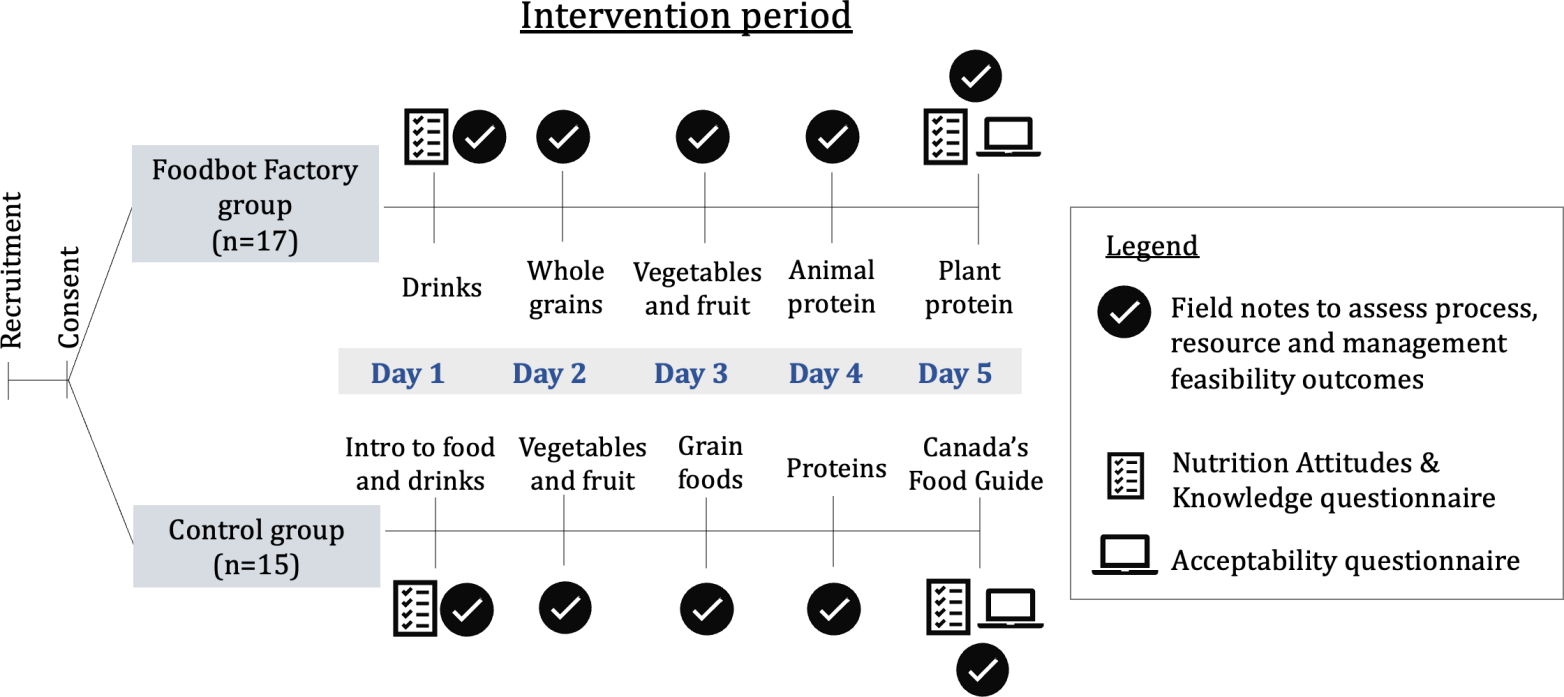
Feasibility study overview*.*

The primary objective was to assess the feasibility, or ability to be successful in implementing the study protocol as planned using feasibility outcomes that are evaluated across 4 domains, that are study processes, resources, management, and a preliminary assessment of scientific outcomes [24]. The specific outcomes within each domain were based on established guidelines for conducting feasibility research [24]. Study process, resource, and management outcomes were documented throughout the study period. The preliminary assessment of scientific outcomes was assessed by having participants complete questionnaires on their nutrition knowledge (day 1 and day 5) and intervention acceptability (day 5; Figure 1).

### Ethical Considerations

This study was approved by the Ontario Tech University Research Ethics Board (#16930) and by the participating school board. Informed consent (Multimedia Appendix 1) was obtained from participants’ parents or guardians, along with informed assent from child participants. All study data are in a deidentified format. Classroom teachers received a $50 CAD (US $37.50) gift card as an acknowledgment for having their classroom participate in the research. All children in the classroom (regardless of participation in the study) received an Ontario Tech University water bottle as a token of appreciation.

### Participants

Grade 4, 4/5, and 5 classrooms in a Greater Toronto Area school board were eligible to participate if they had not yet covered the healthy eating component of the curriculum. Furthermore, 4 classrooms from 2 elementary schools participated in the study. The research team randomly selected schools and contacted them via email to gauge interest in participation. Principals who expressed interest subsequently invited classroom teachers to participate in the study. In each school we recruited 2 classrooms, assigning 1 to the Foodbot Factory group and the other to the control group. Classrooms, rather than individuals, were assigned to the group as the intervention is designed for classrooms and it is not logistically feasible to assign individuals within the same classroom to different study groups. In the first school, 1 classroom was randomly assigned to their group and the second classroom was assigned to the opposite group. In the second school, classrooms were assigned to the study groups based on enrollment to ensure a comparable number of individual participants in each group. Before the study, classroom teachers emailed parents the web-based consent form. Child assent was obtained on day 1 of the study (Monday) from those with parental consent. For participants who were absent on day 1, assent was collected on day 5 for the purposes of collecting data on intervention acceptability only. All children in a classroom participated in the intervention, but only those with parental consent participated in data collection and analysis.

### Interventions

#### Overview

Both interventions were 5 consecutive days in duration and classrooms received nutrition education for 35‐40 minutes per day [23]. The interventions were provided by a hired certified teacher, who was part of the research team (“study teacher”). Due to the nature of their role, the study teacher was not blinded to the intervention group. The study teacher was trained to follow standardized operating procedures for the delivery of the interventions to reduce sources of bias and ensure a high level of fidelity. The study teacher was accompanied by an observer who was trained in the same procedures. The research team originally estimated that data collection would take a maximum of 20 minutes, and that lesson plan delivery would take 40 minutes. Classroom teachers were not involved in teaching the lessons, but they were present in the classroom during the study and supported classroom management.

#### Foodbot Factory Intervention

Classrooms in the Foodbot Factory group received nutrition education using the Foodbot Factory serious game and corresponding curriculum-based lesson plans [19,25]. The serious game is based on experiential learning theory [26], while the lesson plans are rooted in constructivist learning theory, to help children connect knowledge learned in the game to their lived experiences [27]. The Foodbot Factory intervention consists of 5 nutrition education lessons, with additional instructions for teachers on the importance of using food-neutral language, suggestions to incorporate cultural foods into each lesson and modifications for different learning needs. Each lesson follows the 3-phase lesson structure, which is an effective format for structuring a lesson [28]. The first phase of the lesson, “Minds On”, introduces children to the lesson topic and establishes expectations (5‐10 min). In the second phase, “Exploration”, children play through 1 module of the Foodbot Factory serious game on a tablet provided by the research team (10‐15 min). For the final phase, “Consolidation”, discussion and teacher-led activities allow children to connect their new knowledge from the serious game to their previous knowledge and lived experiences (10 min).

#### Control Intervention

Classrooms in the control group received nutrition education that covers the same topics as the Foodbot Factory intervention but used nontechnology based learning materials (eg, activity sheets). The control intervention also consists of 5 nutrition education lessons, following the same 3-phase lesson structure (Table 1). However, in place of the Foodbot Factory serious game, the lesson plans incorporated pre-existing resources that were sourced from a popular website repository of educational materials [29]. These resources were carefully reviewed and selected by the research team to closely match the Foodbot Factory intervention learning goals and ensure their alignment with curriculum and quality. Unlike clinical practice, where there are often existing guidelines for usual care that may serve as a control group in research, there is great variation in the strategies teachers implement for nutrition education. However, a consistent control intervention is required for comparative research purposes. The approach for the control intervention in this study was informed by qualitative interviews and focus groups conducted with Canadian elementary school teachers who frequently reported using pre-existing resources found online for nutrition [10].

**Table 1.** Foodbot Factory and control intervention learning topics and activities.

Daily learning topic and study group		Phase 1: “Minds On” introductory activities and set expectations (approximately 10 min)	Phase 2: “Action” main lesson activity (approximately 10-15 min)	Phase 3: “Consolidation” summarize and review lesson (approximately 10 min)
Day 1—drinks (Foodbot Factory) and introduction to food and drinks (control)				
	Foodbot Factory	Introductory slide show on food and drinks Teacher-led class activity Class discussion	Play “Drinks” module in Foodbot Factory serious game	Complete phase 1 class activity, adding to it based on what was learned Class discussion
	Control	Introductory slide show Complete activity sheet on food groups and drinks independently Class discussion	Complete activity sheet on different food groups and drinks independently	Think-pair-share activity sheet Take up answers from phase 2 activity Class discussion
Day 2/3^a^—whole grain foods (Foodbot Factory day 2 and control group day 3)				
	Foodbot Factory	Introductory slide show Class discussion	Play “Whole Grain Foods” module in Foodbot Factory serious game	Small-group activity and take up answers Class discussion
	Control	Introductory slide show Class discussion	Complete activity sheets on whole grain foods independently or in pairs	Take up answers from phase 2 activity Class discussion
Day 2/3—vegetables and fruit (Foodbot Factory day 3 and control group day 2)				
	Foodbot Factory	Introductory slide show Complete activity sheet on vegetables and fruit independently Class discussion	Play “Vegetables & Fruit” module in Foodbot Factory serious game	Complete phase 1 activity sheet, adding to it based on what was learned Class discussion
	Control	Introductory slide show Class discussion	Complete activity sheets on vegetables and fruit in pairs or small groups	Take up answers from phase 2 activity Complete activity sheet in pairs or small groups
Day 4—animal protein (Foodbot Factory) and protein foods (control)				
	Foodbot Factory	Introductory slide show Class discussion	Play “Animal Protein” module in Foodbot Factory serious game	Teacher-led class activity Class discussion
	Control	Introductory slide show Class discussion	Complete activity sheets on animal and plant protein foods independently or in pairs	Take up answers from phase 2 activity Small group activity sheet Class discussion
Day 5—plant protein (Foodbot Factory) and Canada’s Food Guide (control)				
	Foodbot Factory	Introductory slide show Class discussion	Play “Plant Protein” module in Foodbot Factory serious game	Teacher-led class activity Class discussion
	Control	Introductory slide show Class discussion	Complete activity sheet on Canada’s Food Guide independently	Take up answers from phase 2 activity Class discussion

a Study groups cover both the topics of “Whole Grain Foods” and “Vegetables and Fruit” on different days.

### Outcomes

We holistically determined if each study outcome was feasible for a research protocol by considering the context of each individual outcome, and if the outcome would reasonably facilitate a fully powered study.

#### Study Processes

Feasibility of study processes refers to the main elements of a study that are necessary for its success, including recruitment and retention of participants [24]. We assessed 7 variables related to study processes including school recruitment, classroom recruitment, parent recruitment, child recruitment, attrition rate, data collection, and instrument collection completion rates. Recruitment rates were calculated as the number of recruited school or participants over the total number of contacted or eligible participants (eg, school recruitment rate=schools successfully recruited/schools contacted). The attrition rate was calculated as the number of children recruited to the study with data available from 2 data collection questionnaires, a baseline Nutrition Attitudes and Knowledge (NAK) questionnaire and postintervention NAK, over the total number of child participants. Furthermore, 2 data collection completeness rates were calculated. The first was calculated as the number of child participants with 2 fully complete NAK questionnaires over the total number of child participants who completed the study. The second data collection completion rate was determined by dividing the number of structured field notes completed by study staff by the total number of expected field notes. The instrument collection completeness rate was calculated as the number of child participants with all study questionnaires completed (2 NAKs and the acceptability questionnaire) over the total number of child participants.

#### Study Resources

Feasibility of study resources refers to the time and resources required to run the study [24]. Furthermore, 3 variables related to study resources were assessed including time taken to deliver the intervention, time taken to collect data, and impacts on study personnel who were the study teacher and outcome assessor (ie, what was the qualitative experience for those conducting the study). Variables related to time were taken with a stopwatch and impacts on study personnel were assessed qualitatively. This study did not assess outcomes related to material resources as all necessary resources were brought into classroom by the study team (eg, tablets to play the Foodbot Factory game, activity sheets, and writing supplies).

#### Study Management

Feasibility of study management assesses possible issues with study implementation [24]. In this feasibility study, we collected data on 2 variables related to study management, which included challenges with data collection and challenges with intervention delivery. These outcomes were assessed qualitatively.

#### Preliminary Assessment of Scientific Outcomes

A preliminary assessment of the scientific outcomes of the study included an evaluation of the impacts of the intervention on nutrition knowledge and its safety. These data are intended to inform a fully powered future study, not to determine the effectiveness of an intervention [24]. In total, 3 variables were assessed in this category including acceptability of the intervention, adverse events, and impacts of the intervention on nutrition knowledge. An acceptability questionnaire assessed the perceived acceptability of the intervention by participants. The acceptability questionnaire was based on the theoretical framework of acceptability [30]. The questionnaire was adapted from the generic theoretical framework of acceptability questionnaire to be specific to the nutrition education interventions and ensure readability for children [31]. It consisted of 11 items on a numerical 5-point Likert scale questions defined by level of agreement (1=strongly disagree to 5=strongly agree). Adverse events were documented in study field notes and a self-report question on the acceptability questionnaire asking participants about feelings of stress due to the intervention. Nutrition knowledge was measured using the validated NAK questionnaire, which was developed and validated by our research team and is sensitive to detecting changes in nutrition knowledge [22,32]. The NAK questionnaire consists of 20 questions to assess a child’s overall nutrition knowledge, and 4 subscores of nutrition knowledge (5 questions per subscore) to assess knowledge of a specific food group (eg, drinks, whole grain foods, vegetables and fruit, and protein foods). NAK questions are guided by the recommendations in Canada’s Food Guide [20], which also are part of the Foodbot Factory intervention.

#### Outcome Assessment

Feasibility outcomes were collected throughout the study from (1) structured daily field notes that were created and completed by the research team to document the aforementioned study process, resource, and management outcomes; (2) assessments of nutrition knowledge using the NAK questionnaire [32]; and (3) an acceptability questionnaire. The 2 questionnaires were used to provide a preliminary assessment of the scientific impacts of the intervention.

Field notes were completed each day of the study by the study teacher and observer (days 1 through 5). The NAK questionnaire was administered to participants by an outcome assessor on day 1, before the first nutrition education lesson, and again on day 5, after the final lesson. The acceptability questionnaire was also administered by the outcome assessor and completed on day 5, after participants completed the NAK questionnaire. The questionnaires were completed independently by participants at their desks in the classroom with the outcome assessor circulating to monitor progress. The outcome assessor was present in each classroom throughout the intervention period as an observer, thus they were not blinded to the intervention group. In the first and second classrooms, students completed electronic forms using Qualtrics (Silver Lake); however, there were several issues with electronic data collection (described in Results) and the remaining 2 classrooms completed paper-based forms.

### Sample Size

Our research team previously conducted a pilot study of the Foodbot Factory serious game, which provided us with an understanding of the distribution and effects sizes for the outcome of nutrition knowledge [22]. Thus, our objective with this feasibility study was pragmatic in that we needed to understand the logistics of recruitment, data collection, and intervention delivery in the classroom setting [33]. For this type of feasibility study, 12 to 30 participants per group is suggested by other researchers [34,35]. Therefore, we aimed to recruit 4 classrooms as the feasibility challenges in this study were likely to arise related to both the classroom environment and individual participants. This resulted in 102 children eligible to participate and allowed us to assess the feasibility of implementing the interventions in different neighborhoods and understand the pragmatic elements required to scale the intervention up in a fully powered study.

### Data Analysis

Outcomes related to study processes were analyzed using descriptive statistics, frequencies, and percentages. Resource outcomes were assessed using descriptive statistics and impacts on study personnel were assessed narratively. Outcomes related to management were assessed narratively. The scientific outcome of acceptability was analyzed using descriptive statistics. On all Likert scale questions, a response of 1 or 2 was considered as disagreement, a response of 3 was considered neutral, and a response of 4 or 5 was considered as agreement. Intervention safety was analyzed using both descriptive statistics and narratively. The scientific outcome of intervention impacts was assessed using descriptive statistics but was not considered in the final determination of protocol feasibility as the sample size would not have sufficient power. Within-group analysis for changes in overall nutrition knowledge and nutrition knowledge subscores was completed using a paired *t* test, after confirming the data were normally distributed through the Shapiro-Wilk test. Participants were excluded from the analysis of the overall knowledge score if there were any missing data. However, participants were included in nutrition knowledge subscore analyses if they had complete data for a particular subscore. All statistical analyses were completed using R Statistical Software version 4.2.0 (R Core Team) [36].

## Results

### Overview

**Figure 2.**
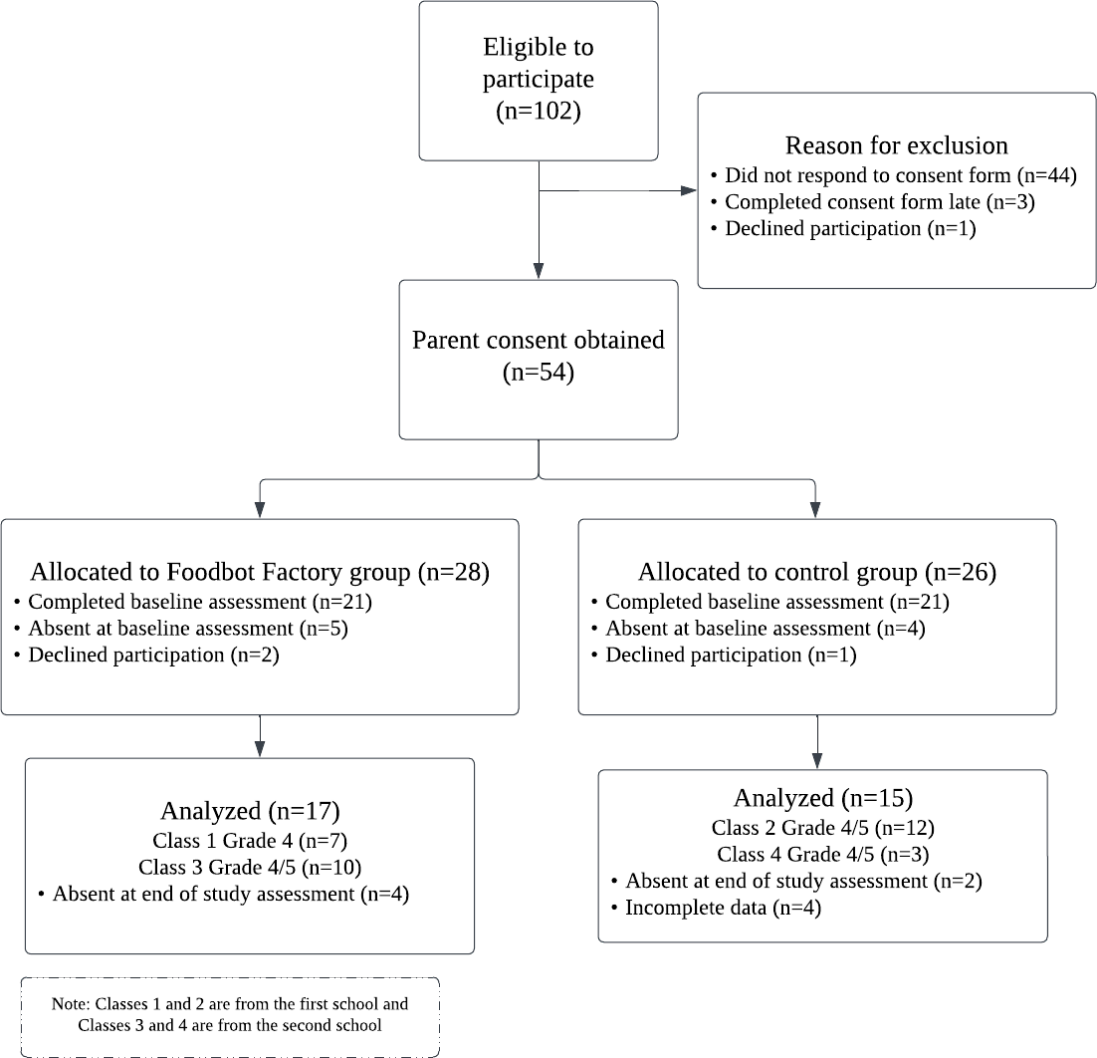
Flowchart of participant progress through the study.

In total, 4 classrooms participated from 2 schools between February and May 2023 (Figure 2). Both schools were in urban areas in culturally and linguistically diverse neighborhoods. The first school was in a higher socioeconomic-status neighborhood while the second school was in a neighborhood with a higher proportion of newcomers to Canada and government-subsidized housing. Participating classrooms included one Grade 4 and three Grade 4/5 split classes.

### Study Process Outcomes

The school recruitment rate was low (3/20, 15%). Of the 20 principals who were contacted by email, 70% (n=13) did not respond, 10% (n=2) declined participation, and 5% (n=1) replied with a question about the study but lost to follow-up. To counter the low email response rate, the research used phone calls for recruitment and was able to recruit 1 school after 3 phone calls. Once a principal agreed to have their school participate, 100% of classroom teachers in those schools agreed to have their classroom participate. In total, 4 classrooms from 2 of the 3 recruited schools were scheduled for participation, with a total of 102 children. While a third school was recruited to participate, they ultimately were not scheduled to be in the study, due to a staffing shortage among the research team. Parent recruitment rate was low (54/102, 53%); 43% (44/102) did not respond to the consent form, 3% (3/102) consented after the study start date, and 1% (1/102) declined participation. To improve parent recruitment, the research team requested that classroom teachers send a reminder email. Reminders were sent by teachers in 2 classrooms, resulting in a 77% parent recruitment rate compared with 40% in classrooms with no reminders. Anecdotally, 1 classroom with lower parent recruitment had a high number of parents who did not speak English as a first language, and the classroom teacher noted this was likely a barrier to participating in the study. Most children with parental consent (49/54, 91%) agreed to participate in the study, with 24 in the intervention group and 25 in the control group. The average age of participants was 9.6 (SD 0.68) years. Participants self-reported their gender as a boy (24/49, 49%), girl (18/49, 37%), or a self-defined identity (2/49, 4%), with the remaining participants preferring not to answer (5/49, 8%). In the intervention group, 17 participants completed the study; however, only 10 had a complete set of data. In the control group, 15 participants completed the study, with 9 having a complete set of data. The attrition rate was 19%. For participants, the instrument collection completion rate was moderate (73%) and the data collection completion rate for the NAK questionnaire was low (59%). The data collection completion rate of the structured field notes by study staff was 100%.

### Study Resource Outcomes

The average time required for child assent was 6.5 minutes, lesson plan delivery was 34 minutes (8 min for minds on, 14 min for exploration, and 12 min for consolidation), acceptability questionnaire completion was 5.5 minutes, and NAK questionnaire completion was 13 minutes; all consistent with original estimates. Study personnel, including the study teacher and outcome assessor, reported an overall positive experience as they enjoyed interacting with the students. In one classroom, classroom management was a significant challenge due to a handful of students who frequently interrupted the lessons and outcome assessments. This was a minor psychological stressor for the study teacher. There were challenges with retaining the hired study teachers on the project due to career changes and an unanticipated emergency, requiring the research team to reschedule 2 classrooms to a later date.

### Study Management Outcomes

Data collection procedures and delivery of the intervention were executed as planned, although some challenges were experienced. Tablets were originally used for participants to complete data collection forms, but this posed a few unanticipated challenges. For example, participants intentionally or unintentionally “refreshed” or navigated away from the web-based NAK questionnaire, leading to the loss of completed responses. In addition, once a NAK questionnaire was submitted, study personnel were unable to verify if all questions had been completed. This resulted in incomplete data for some participants. Several participants struggled to focus on completing the data collection forms due to distractions in the classroom. The most significant distraction occurred when other children who were not participating in the study were allowed by the classroom teacher to engage in other activities, namely the use of electronic devices. Furthermore, as participants completed the questionnaire independently, those who finished early then moved on to a different activity. Participants who needed more time to complete the questionnaire became increasingly distracted as others around them finished, which may have contributed to them rushing to finish and skipping questions.

Based on the implementation of the interventions in the first 2 classrooms, some changes were made to the intervention delivery that were implemented in the subsequent 2 classrooms. The changes clarified instructions, modified select discussion questions to improve wording and added slideshows with the daily learning objectives instead of writing them on the board. Overall, these changes were relatively minor and were intended to improve the clarity and accessibility of the lesson delivery. In the study, classroom management was particularly challenging in 1 classroom due to some children repeatedly interrupting the study teacher. Study personnel found that having the classroom teacher and educational assistants support classroom management was helpful. In 2 classrooms, the intervention schedule was modified to occur over 4 days instead of 5, due to a conflicting school-wide track and field event. On one of the 4 days, 2 intervention lessons were provided consecutively instead of having 1 lesson each day. While it was logistically feasible to provide 2 lessons back-to-back in 1 day, student engagement was lower during the second lesson. Another challenge occurred in the Foodbot Factory intervention group, where some children struggled with the augmented reality components in the Foodbot Factory serious game. The research team responded to this by improving instructions about how the augmented reality features work. In addition, some children struggled to transition away from using the tablet to play the Foodbot Factory serious game to the next learning activity as they found the game engaging and wanted to keep playing.

### Preliminary Assessment of Scientific Outcomes

**Figure 3.**
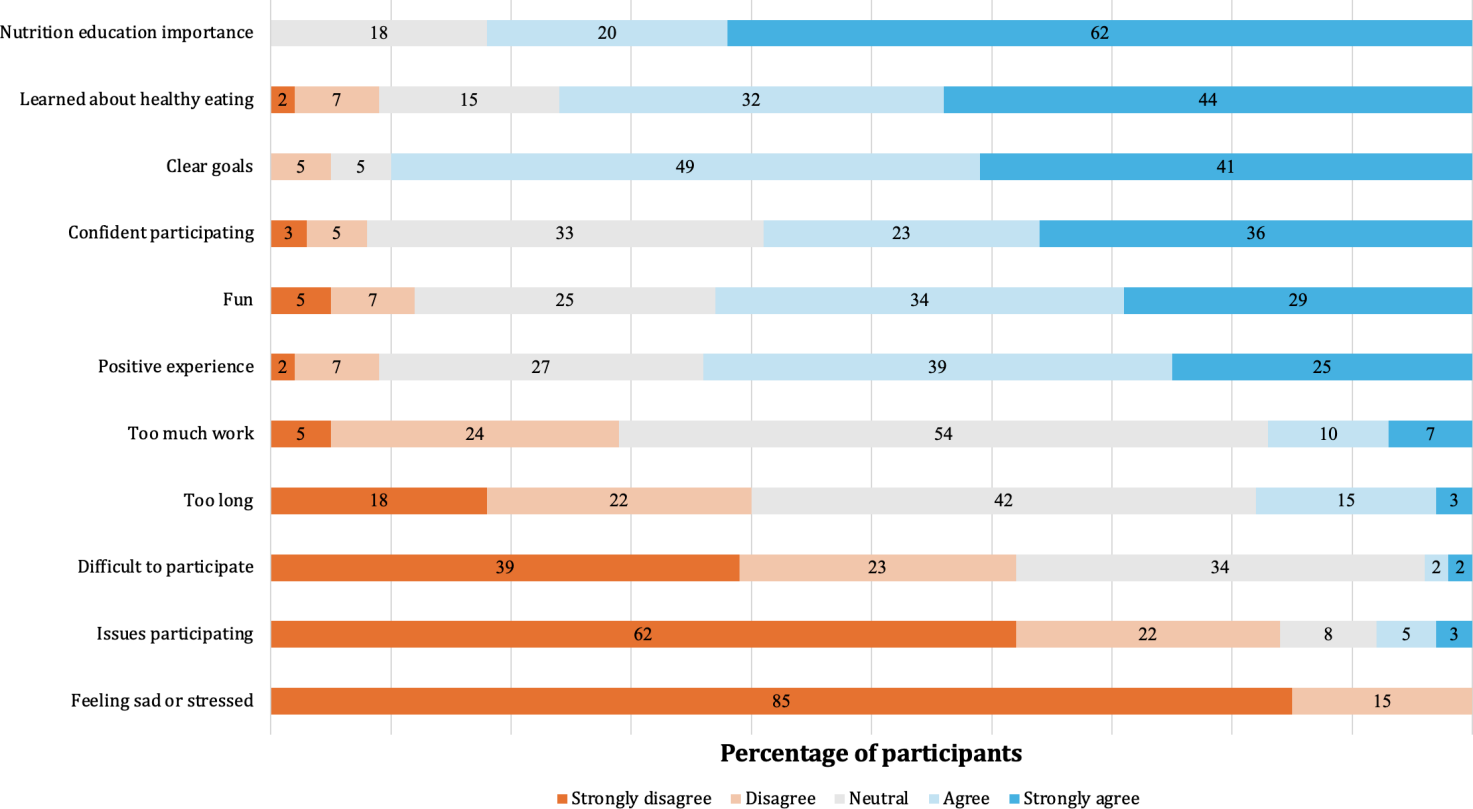
Acceptability of the nutrition education interventions as rated by participants (n=41).

Based on observations, participants enjoyed participating in both the Foodbot Factory and control intervention lessons and were engaged in learning about nutrition through the activities. Overall, intervention acceptability among participants was moderate to high, with most participants reporting that they had a positive experience (26/41, 63%), understood the goals of the nutrition education lessons (37/41, 90%), and that they learned something new (31/41, 76%; Figure 3). No participants reported feeling sad or stressed about food and nutrition from the lessons on the acceptability questionnaire nor did study personnel observe any verbal or nonverbal signs of concern from children in the classroom.

Scores on the NAK questionnaire were normally distributed as determined by the Shapiro-Wilk test. Both groups demonstrated improvements in their overall nutrition knowledge (Foodbot Factory group: mean 10.8, SD 2.1 to mean 12.5, SD 3.5; control group: mean 11.8, SD 1.9 to mean 12.7, SD 1.8), although these improvements were not statistically significant. For subscores of nutrition knowledge, participants in the intervention group demonstrated statistically significant improvements in knowledge of vegetables and fruit and protein foods. Participants in the control group demonstrated statistically significant improvements in knowledge of grain foods (Table 2).

**Table 2.** Changes in overall and subscores of nutrition knowledge within the Foodbot Factory and control groups^a^.

Nutrition knowledge	Foodbot Factory group				Control group			
	Score, n	Baseline, mean (SD)	End of study, mean (SD)	*P* value	Score, n	Baseline, mean (SD)	End of study, mean (SD)	*P* value
Overall nutrition knowledge	10	10.8 (2.1)	12.5 (3.5)	.13	9	11.8 (1.9)	12.7 (1.8)	.09
Drinks	17	3.94 (0.66)	3.76 (0.56)	.33	15	3.93 (0.70)	3.93 (0.59)	≥.99
Grain foods	16	2.31 (1.2)	2.75 (1.1)	.17	15	2.87 (1.1)	3.40 (1.1)	.03
Vegetables and fruit	15	2.27 (0.88)	3.00 (0.93)	.04	14	2.86 (0.86)	3.07 (1.0)	.61
Protein foods	12	1.92 (0.90)	2.75 (1.6)	.03	10	2.60 (1.1)	1.70 (0.95)	.05

a Data presented as means and SDs with paired *t* tests used to assess changes in knowledge from baseline to the end-of-study within groups. Sample sizes vary as only participants with a complete set of responses were included in the analysis.

## Discussion

### Principal Findings

Our research study determined the feasibility of implementing the Foodbot Factory nutrition education intervention as part of a research protocol in Grade 4 and 4/5 classrooms, identifying 9 facilitators to support a larger study and 4 risks that will require mitigation. Elements of the study that were deemed feasible included classroom recruitment, child recruitment, the attrition rate, time taken to deliver the intervention and collect data, the impacts of the study on personnel, management of delivering the intervention, lack of adverse events, and acceptability of the intervention. However, strategies will be needed to improve school recruitment, parent recruitment, data collection completeness, and the management of data collection procedures. In this feasibility study, we made minor modifications to the protocol on implementation to improve recruitment and intervention implementation (eg, conducting recruitment phone calls and modifying intervention instructions for the classroom). With further modifications to mitigate the aforementioned risks, our findings largely support the feasibility of this research protocol to evaluate the Foodbot Factory nutrition education intervention as part of a fully powered randomized controlled trial.

The primary strength of this study was our ability to better understand how to collaborate with schools, teachers, parents, and children in our future research. Key takeaways learned from this study that will be valuable to other researchers, include the importance of consulting with school boards before conducting research, ensuring recruitment materials and methods are relevant for the target audience, engaging classroom teachers and testing data collection methods before their implementation. We discussed our methodology and objectives with staff from several school boards before seeking school board approval to conduct the research. From the outset, this approach resulted in a protocol that would be more feasible and acceptable to implement in classrooms and ensure value to our participants and the school board. For example, the initial version of our study protocol requested 5 hours of total class time, which was perceived as too much time taken away from other curriculum. Not only did we modify the protocol to reduce the total time by 1 hour, we also updated our recruitment materials to highlight alignment of the study with curriculum. This was a critical change as teachers have very limited time to cover content that is outside of the curriculum. Ultimately, the resulting protocol is not only acceptable to school boards, but the duration of the intervention now better aligns with the time teachers typically allocate to a lesson and clearly communicates the value of the study to possible participants. These features increase real-world acceptability of the intervention. Researchers should consult with their local school boards and teachers to establish a collaborative relationship from the beginning to develop interventions and research protocols that are suitable and valuable.

When recruiting individual schools, classroom teachers, and parents, recruitment materials should be succinct and highlight the value of study participation. In this study, phone calls were effective at recruiting schools as they enable direct consultation on the practical aspects of the study and allow the school to quickly evaluate if the study will work for them. Low recruitment of schools was still seen in this study and has been reported as a challenge in other school-based health studies due to low interest, poor timing, and other commitments [37,38]. During this study, engagement of the classroom teacher was critical for parent recruitment, as we were not permitted to contact parents directly. We found low parent recruitment was driven by parents not returning consent forms, rather than them explicitly declining participation, which has been observed in other studies [37]. We also observed significant improvements in recruitment when the classroom teacher sent reminders to parents about the study, highlighting the important role of classroom teachers in engaging parents in study participation. In this study, classroom teachers were asked to contact all parents and guardians to share the consent form and reminders to complete it. However, classroom teachers may introduce selection bias if they selectively reach out to parents and guardians. Instructions for sending out parent consent forms to classroom teachers should be clear and concise to avoid this bias.

In this study, we show the importance of testing data collection methods before implementation in a classroom setting. First, an important takeaway from this study is that electronic data collection presented several challenges. Although other studies have used electronic data collection methods, it may present more challenges if the technology has not been used before in that context [39]. In this study, training was not provided to participants on how to complete the electronic data collection form before data collection as we did not have sufficient time allotted for such training. Training on data collection methods could minimize issues like participants accidentally refreshing the page and skipping questions [39]. In this study, questions on the electronic data collections forms were not made mandatory for submission as study participation was voluntary. Second, participants completed data collection independently, requiring them to use their literacy skills to read and respond to each question. This data collection approach reduced accessibility and led to participants finishing at varying time points and the classroom environment becoming increasingly distracting for participants who needed more time. Missing data from both missed responses and missing surveys in other school-based studies is a common phenomenon ranging from 11%‐51% at a given outcome assessment time point [40,41]. In a future protocol, data collection could be modeled on the procedures classrooms use when administering standardized tests. This would align research data collection with procedures that are already familiar to children, provide clear instructions, increase accessibility, allow for verification of data collection completeness to encourage complete responses and directly guide the classroom as a group through the questions so all participants finish at the same time.

This study also examined the impacts of the developed nutrition education interventions on children’s nutrition knowledge. Statistically insignificant improvements in overall nutrition knowledge were observed in both groups, and participants in the Foodbot Factory group significantly improved their knowledge of vegetables and fruit and protein foods. The results within-groups at the individual level indicate improvements in nutrition knowledge but do not have the same effect size as a larger proof-of-concept study of the Foodbot Factory serious game, where significant improvements in children’s overall nutrition knowledge were observed both within and between groups [22]. This indicates that the findings in this feasibility study for nutrition knowledge are inconclusive, due to the insufficiently powered sample size, and should be used primarily for descriptive purposes and informing improvements to the intervention [42]. Some changes were made to the lesson plans during the study to improve clarity and accessibility (eg, using a slideshow to share learning objectives instead of writing them on the board) as the process of conducting feasibility studies and developing research protocols is recognized to be iterative and adaptive [43]. Since these changes were relatively minor and were focused on delivery, rather than intervention content, we do not anticipate they influenced the outcomes of interest.

This study had several strengths and limitations that can inform future research. This study included a small sample size, which is primarily driven by low parent recruitment. Recruitment may be a primary feasibility challenge moving forward to a fully powered study. Including more classrooms may have revealed additional feasibility issues; however, the classrooms were intentionally sampled from schools in neighborhoods with known differences in their sociodemographic profiles. While the challenges in this study are similar to those observed in international research [37,38,40,41], the results may be most applicable for those conducting school-based research in the Greater Toronto Area, or other urban environments in Ontario, Canada. In this study, we chose not to randomize classrooms as we were unsure of how successful our recruitment would be and this approach would allow us to ensure balance between study groups. Nonrandomized feasibility studies are common for research in earlier phases of preparation for a trial and when there are significant unknowns [44]. To address the lack of randomization and balance school- and neighborhood-level characteristics, the research team assigned 1 classroom at each school to a different group; however, we acknowledge the potential of selection bias and an uneven balance of characteristics between groups that may have been introduced. In future research, we will recruit 2 classrooms per school and use pair-matching randomization to both reduce bias and ensure groups are balanced on school and neighborhood level variables that can impact our outcomes of interests. Due to the study design, where classrooms rather than individuals were assigned to each treatment arm, we were unable to assess differences between groups as the data should be analyzed at the classroom cluster level, which this study was not powered to do. In future research, an adequately powered randomized controlled trial that randomizes classrooms as clusters would be more appropriate to assess the impact of the interventions in a pragmatic fashion. In this study, we provided all material resources necessary, including tablets for students to use the Foodbot Factory serious game. This can be considered as a strength, as it increased the feasibility of conducting the research, but also a limitation as we did not evaluate possible material resource limitations (and the knowledge and processes required to implement them) for conducting technology-based research in schools. Researchers should carefully consult with school boards to understand the availability of resources to ensure success or invest in the required technology to avoid material resource barriers as we have done in this study. We also chose to use a certified study teacher who was trained to implement the lesson plans as it increases feasibility from the school board. We will use this approach in a future efficacy study. Future research should aim to also evaluate the effectiveness of the research when the lesson plans are implemented by classroom teachers. At this stage, our research team did not assess retention of knowledge or examine other variables that may be impacted by a nutrition education intervention, including dietary intake and behaviors. We also did not assess acceptability of the study protocol from the perspective of classroom teachers, which was not the focus of this study. Retention of knowledge and dietary intake will be assessed in future research and qualitative interviews will also be conducted with classroom teachers to inform implementation needs and strategies for the Foodbot Factory intervention. A current limitation of the Foodbot Factory intervention is that it predominantly covers nutrition knowledge and does not address all elements of food literacy. The serious game is also only available for use on mobile devices. Our future work aims to make a web-accessible version of the Foodbot Factory serious game and expand the content to different age groups and food literacy concepts.

### Conclusions

In conclusion, the majority of feasibility outcomes assessing study processes, resources, management, and intervention acceptability suggest that the study protocol was feasible [45]. The data offer several insights to inform future studies and, when the protocol is implemented into a fully powered trial, the successes identified can be leveraged, and the risks associated with school recruitment, parent recruitment, and data collection can be averted with targeted mitigation strategies. These lessons learned have been applied to our protocol for a fully powered cluster randomized controlled trial evaluating the efficacy of Foodbot Factory in classrooms [46]. The development and evaluation of the protocol in this study was an important step in understanding how to best evaluate technology-based nutrition education interventions in Canadian classrooms. The lessons learned in this study will also support the rising number of researchers embarking on school-based nutrition and health interventions in school and classroom settings both in Canada and abroad.

## Supplementary material

10.2196/69242 Multimedia Appendix 1 Informed consent form.

## Abbreviations

NAK: Nutrition Attitudes and Knowledge
