# Supplementary material for: Evaluation of a Curriculum-Based Nutrition Education Intervention Protocol in Elementary Schools: Nonrandomized Feasibility Study
Source: JMIR Form Res. 2025 Apr 16;9:e69242. doi: 10.2196/69242 (PMC12016673; doi:10.2196/69242)
Supplement: Multimedia Appendix 1 [file formative-v9-e69242-s001.docx]

**Consent Form to Participate in a Research Study**

**Title of Research Study:** Foodbot Factory Pilot Study

**Name of Principal Investigator (PI):** Dr. JoAnne Arcand

**PI’s contact number/email:** joanne.arcand@ontariotechu.ca

**Co-Investigator and email:** Dr. Ann LeSage ann.lesage@ontariotechu.ca

**Student Lead and email:** Jacqueline Brown jacqueline.brown@ontariotechu.ca

**Departmental and institutional affiliation:** Ontario Tech University Faculty of Health Sciences

**Introduction**

We are inviting your child to participate in a research study entitled “Foodbot Factory Pilot Study”. Your child is being asked to take part in a research study. Please read the information about the study presented in this form. The form includes details on the study’s procedures, risks and benefits that you should know before you decide if you would like your child to take part. You should take as much time as you need to make your decision. You should ask the Principal Investigator (PI) or study team to explain anything that you do not understand and make sure that all of your questions have been answered before signing this consent form. Before you make your decision, feel free to talk about this study with anyone you wish including your friends and family. Participation in this study is voluntary.

This is a third-party study/research project and not a TDSB study/research project. This study

has been reviewed by the Ontario Tech University Research Ethics Board REB [#16930] on

[09/12/2022]. TDSB External Research Review Committee has granted approval for this

study/research project. The school principal has also given permission for this study/research

project to be carried out in this school.

**Purpose and Procedure**

*Purpose*

We are teachers and researchers at the Ontario Tech University. We are working with your child’s school board and classroom teacher to test different ways to teach children about food and nutrition. Your child has been invited to participate in this study because their classroom teacher has agreed to allow our team to test different formats of nutrition education with their students.

We are asking for your consent for your child to participate in our research project. If you do provide consent, your child will also be asked for his/her/their assent before participating in this study in their classroom. Assent means expressing approval or saying yes to participating.

*Procedure*

As part of this **voluntary research study**, we will test two ways of teaching children about nutrition in two different classrooms. Your child’s class will be randomized (like the flip of a coin) to receive one of the two types of nutrition education. Regardless of which classroom your child is in, the class will receive their nutrition education from an Ontario Tech University certified teacher (not their usual classroom teacher). This project has been informed by experts in dietetics and education, to create fun and interactive activities for nutrition education. In order to understand which educational strategies are best for students’ learning, we need information on how students’ nutrition knowledge and attitudes change by participating in the nutrition education activities.

The educational activities we have developed will help with the teaching and learning of nutrition curricula, with an emphasis on healthy eating messages and nutrients that promote health. Daily nutrition education sessions will be 35-40 minutes/day for each of the 5 days. Your child will be asked to participate in various nutrition education activities throughout the week. Information will be collected from you and your child(ren) at various times during the project. Information is collected using secure online forms administered using Qualtrics online software. We will gather information in the following ways:

- We would like your child(ren) to answer some questions about their knowledge of and attitudes towards healthy eating using a short questionnaire. We would like your child(ren) to answer these questions 2 times: before, and immediately after the nutrition education activities. Each time, this questionnaire will take approximately 7 minutes to complete.
- On the last day of the nutrition education activities, we would like your child(ren) to answer some questions about the acceptability of their learning experience (i.e., enjoyment of the activities, level of difficulty) with the nutrition education activities they completed in the classroom using a short questionnaire. This questionnaire will take approximately 5 minutes to complete.

Data will only be collected and analyzed from children who have parental consent and who have provided assent to participate in the research study. Findings from this project may be published in journals and presented at conferences and will inform further research on how to help children learn about nutrition in the classroom.

**Potential Benefits**

Potential benefits for participation in this study include: learning through engaging nutrition education activities that align to Canada’s Food Guide and gaining understanding of how food and nutrition impacts health.

**Potential Risks or Discomforts**

Potential risks or discomforts include: feeling pressure to share or embarrassment from sharing work and feeling coerced into participating in the research because the rest of the class appears to be. Risks will be dealt with in the following ways: researchers/teachers will explain to children that they have the right to pass when it comes time to sharing their work. They will also explain that student participation in the research portion of the activities is entirely voluntary and that they may choose not to participate at any point, without penalty.

**Use and Storage of Data**

Data for this study will be in electronic format, collected through the Qualtrics online software and housed securely in deidentified format. This means that it will not contain any information that can identify you or your child, via cloud storage on Google Drive in the Ontario Tech server. The study data, including all electronic files, will be kept in deidentified format for three years, and properly destroyed after this time by erasing the electronic files. Study data will be accessible to the members of the research team (Dr. Arcand, Dr. LeSage and Ms. Brown). We will collect the names of consenting parents and their children for the purposes of consent and assent. This identifiable information will be stored separately on an encrypted and password protected USB key.

**Confidentiality**

All information collected will be strictly confidential and protected in accordance with all

applicable privacy laws, including Municipal Freedom of Information and Protection of Privacy

Act, R.S.O. 1990, c. M.56 (“MFIPPA”) and Toronto District School Board (“TDSB”) policies and

procedures. The researcher, and no one else, is responsible for ensuring the confidentiality of

any information collected during the study/research project. The information you and your child provides will not be stored with personal identifiers, nor will your child be identified in any publications or presentations. Your privacy and your child’s privacy shall be respected. No information about your identity will be shared or published without your permission, unless required by law. Confidentiality will be provided to the fullest extent possible by law, professional practice, and ethical codes of conduct. Please note that confidentiality cannot be guaranteed while data is in transit over the Internet. Due to the nature of this study taking place in a group setting, your child’s classroom teacher and other students in the classroom may be aware of your child’s participation in the study.

**Participation**

Your child’s participation in this study is voluntary and they may partake in only those aspects of the study in which they feel comfortable. You and your child may also decide not to be in this study, or to be in the study now, and then change their mind later. Your child may leave the study at any time without any penalty. You and your child will be given information that is relevant to your decision to continue or withdraw from participation. Such information will need to be subsequently provided.

Children should not feel obligated or compelled to participate in the research for any reason. Participation in the research is entirely optional and children will not be penalized in any way if they do not participate. Your child can choose not to answer specific questions.

**Right to Withdraw**

Your child may withdraw from the research study at any time and you do not need to offer a reason for making this request. If your child withdraws from the study, any data that they have contributed will be deleted. If, during any of the above-described activities, your child decides not to participate, they can end involvement in the activity by indicating that they wish to withdraw from the study. Your child may communicate refusal verbally or non-verbally. If you or your child would like to withdraw from the study *after* data collection is completed, please inform us within 4 weeks of study completion.

**Compensation, Reimbursement and Incentives**

You and your child will not incur any expenses as a result of participating in this study. All children in a participating classroom (regardless of participation in the study) will receive an Ontario Tech University branded water bottle.

**Debriefing and Dissemination of Results**

If interested, you can stay informed of the results of the study at our lab website www.arcandnutritionlab.com

**Participant Rights and Concerns**

Please read this consent form carefully and feel free to ask the researcher any questions that you might have about the study. If you have any questions about your rights as a participant in this study, complaints, or adverse events, please contact the Research Ethics Office at (905) 721-8668 ext. 3693 or at [researchethics@ontariotechu.ca](mailto:researchethics@ontariotechu.ca). If you have questions concerning the research study or your child experiences any discomfort related to the study, please contact the study lead Jacqueline Brown at 905.441.1376 or jacqueline.brown@ontariotechu.ca or the Principal Investigator, Dr. JoAnne Arcand at 647.296.8426 or [joanne.arcand@ontariotechu.ca](mailto:joanne.arcand@ontariotechu.ca). By signing this form you do not give up any of your legal rights against the investigators, sponsor or involved institutions for compensation, nor does this form relieve the investigators, sponsor or involved institutions of their legal and professional responsibilities.

Thank you for considering allowing your child to participate in this research study. **If you have more than one child enrolled in the participating classroom, please complete this form for each child.** Please note that your child(ren)’s signature is also required and will be collected in class prior to beginning the study. Both parent and student consent/assent are required to participate in this study.

*Required

Parents/Guardians

Your agreement on the consent forms indicates the following:

I have read the consent form and understand the study being described.

I have had an opportunity to ask questions and those questions have been answered. I am free to ask questions about the study in the future.

I freely consent to have my child participate in the research study, understanding that I or my child may discontinue participation at any time without penalty. A copy of this consent form has been made available to me.

By agreeing below, I consent to have my child participate in this research study.

Student Name: *

Classroom Teacher Name:

Parent/Guardian Name: *

Date: *

I give my permission for my child to participate in the research study*

Yes

No
